# Rerefinement of the crystal structure of tri­chlorido­sulfonium­(IV) hexa­chlorido­uranate(V), (SCl_3_)[UCl_6_]

**DOI:** 10.1107/S2414314620009608

**Published:** 2020-07-17

**Authors:** Holger Lars Deubner, Sergei I. Ivlev, Florian Kraus

**Affiliations:** a Anorganische Chemie, Fachbereich Chemie, Philipps-Universität Marburg, Hans-Meerwein-Strasse 4, 35032 Marburg, Germany; Vienna University of Technology, Austria

**Keywords:** crystal structure, uranium, disulfur dichloride, ion pair

## Abstract

Single crystals of tri­chlorido­sulfonium­(IV) hexa­chlorido­uranate(V) were obtained from the reaction of uranium(IV) chloride with an excess of disulfur dichloride and studied by single-crystal X-ray diffraction. In comparison with the structure model reported previously [Sawodny *et al.* (1983 ▸). *Z. Anorg. Allg. Chem.* **499**, 81–88.], the lattice parameters and fractional atomic coordinates were determined to a much higher precision, leading overall to an improved structure model. The ionic compound contains trigonal–pyramidal (SCl_3_)^+^ cations and slightly distorted octa­hedral [UCl_6_]^−^ anions. The structure was refined as an inversion twin with a twin ratio of 4.4:1.

## Abstract 2

A redetermination of the crystal structure of tri­chlorido­sulfonium­(IV) hexa­chlorido­uranate(V) is described and compared with the previously reported structure.

## Graphical abstract

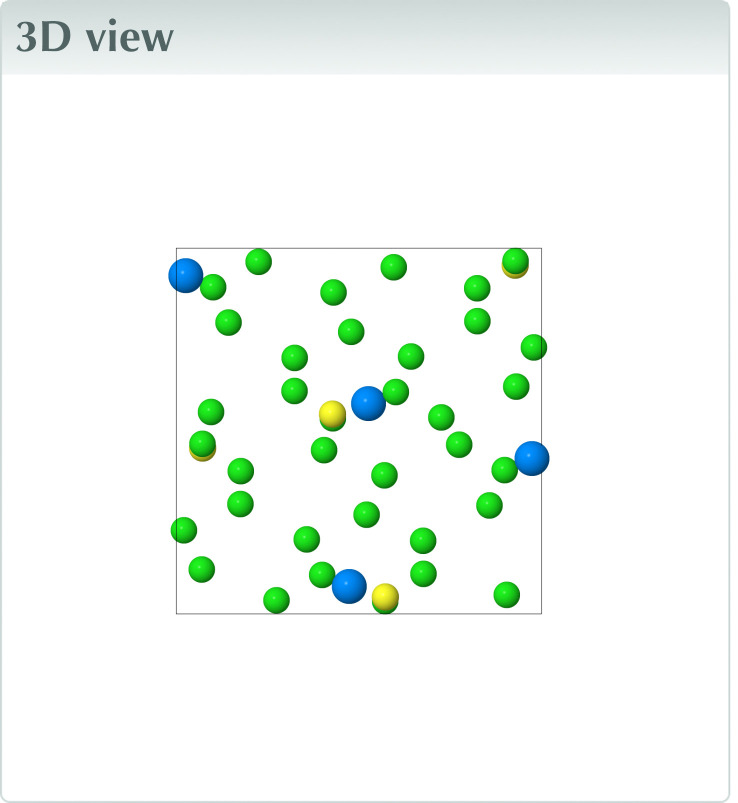

## Structure description

We explored the reaction of uranium tetra­chloride with disulfur dichloride out of curiosity. Moreover, we investigated whether the latter compound could be a potential solvent for uranium halides. During these studies, high-quality single crystals of (SCl_3_)[UCl_6_] were obtained.

The lattice parameters determined at 100 K from the current single-crystal X-ray structure determination (Table 3 ▸) agree with those reported previously [*a* = 10.668 (10), *b* = 10.712 (4) *c* = 11.333 (6) Å at *T* = 293 K; Sawodny *et al.*, 1983 ▸].

**Figure 1:**
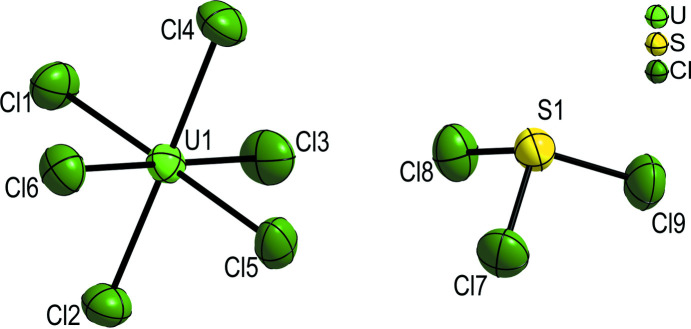
The slightly distorted trigonal–pyramidal [SCl_3_]^+^ cation and octa­hedral [UCl_6_]^−^ anion of the title compound. Displacement ellipsoids are shown at the 90% probability level.

The U^V^ atom is located on Wyckoff position 4 *a* and has six chloride ligands in its slightly distorted octa­hedral coordination sphere (Fig. 1 ▸). The U—Cl bond lengths range between 2.4869 (16) and 2.5209 (14) Å and are in good agreement with the previously reported values [U—Cl distances = 2.485 (11)–2.531 (10) Å; Sawodny *et al.*, 1983 ▸]. A comparison between the U—Cl bond lengths and Cl—U—Cl angles obtained from the current and the previous refinements is collated in Tables 1 ▸ and 2 ▸, respectively. Similar U—Cl distances are observed: (i) in the crystal structure of the low-temperature modification of UCl_6_ where the coordination sphere for the U atom is also distorted octa­hedral but with slightly shorter U—Cl bonds of 2.4443 (15)–2.4570 (20) Å (at 100 K; Deubner *et al.*, 2019 ▸) due to the presence of a U^VI^ atom, and (ii) in Cs_2_[UCl_6_] with longer U—Cl bonds of 2.621 Å (at 293 K; Schleid *et al.*, 1987 ▸) due to the presence of an U^IV^ atom.

The S^IV^ atom is also located on Wyckoff position 4 *a* and has three chloride atoms in its trigonal–pyramidal coordination sphere (Fig. 1 ▸). The S—Cl bond lengths are virtually the same at 100 K. In comparison, Sawodny *et al.* (1983 ▸) reported slightly shorter S—Cl bond lengths for (SCl_3_)[UCl_6_] at 293 K (Table 1 ▸). Nevertheless, these atomic distances are also in good agreement with those reported for the ionic compound *β*-[SCl_3_][SbCl_6_] [1.979 (5) to 1.992 (7) Å at 169 K; Minkwitz *et al.*, 1992 ▸]. The Cl—S—Cl bond angles in (SCl_3_)[UCl_6_] resulting from the current and the previous refinements differ slightly (Table 2 ▸).

**Figure 2:**
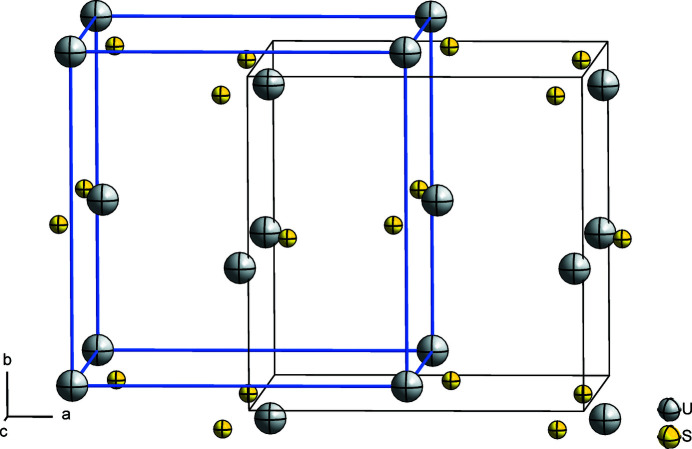
Packing of the U and S atoms in the crystal structure of (SCl_3_)[UCl_6_], showing a distorted NaCl-type arrangement. The unit cell drawn in black can be shifted to the one highlighted in blue to make the relation more easily visible. The idealized blue unit cell shows the deviation of U atoms from *F*-centering as well as the deviation of the (SCl_3_)^+^ entities (Cl atoms not shown) from the octa­hedral voids.

The packing of U and S atoms in the crystal structure of (SCl_3_)[UCl_6_] is shown in Fig. 2 ▸. As can be seen, the U and S atoms are arranged according to a distorted NaCl-type of structure. The overall coordination sphere of the S atom can be regarded as [3 + 3], with the three long S—Cl inter­actions being 3.0721 (2), 3.160 (2) and 3.287 (2) Å. The corresponding coordination polyhedron is a distorted trigonal anti­prism, with the S atom displaced from the center.

## Synthesis and crystallization

(SCl_3_)[UCl_6_] was synthesized in a borosilicate Schlenk tube from uranium tetra­chloride (35 mg, 0.09 mmol) in disulfur dichloride (3 ml) at 358 K over a period of four months. A selected dark-yellow crystal was chosen for single-crystal X-ray diffraction.

We assume that S_2_Cl_2_ disproportionates under the applied reaction conditions and that elemental chlorine, sulfur monochloride, as well as the sulfur chlorides S_3_Cl_2_ and S_3_Cl_4_ are produced in the chemical equilibria described in equations (1)–(3).

3 S_2_Cl_2_ → S_3_Cl_2_ + S_3_Cl_4_ [equation (1); Spong, 1933 ▸].

S_3_Cl_4_ → S_2_Cl_2_ + SCl_2_ [equation (2); Spong, 1933 ▸].

S_3_Cl_4_ → S_3_Cl_2_ + Cl_2_ [equation (3); Spong, 1933 ▸].

Chlorine is dissolved in an excess of S_2_Cl_2_ and may then act as an oxidant oxidizing uranium(IV) chloride to form UCl_5_ [equation (4)]. Other chlorine-sulfur species may also be responsible for the oxidation.

2 UCl_4_ + Cl_2_ → 2 UCl_5_ [equation (4); Cordfunke *et al.*, 1982 ▸].

We further assume that the formed SCl_2_ [equation (2)] may disproportionate to S_2_Cl_2_ and SCl_4_ [equation (5)].

3 SCl_2_ → S_2_Cl_2_ + SCl_4_ [equation (5); Lowry *et al.*, 1927 ▸].

Finally, the formation of the title compound may be described by the reaction of the Lewis acid UCl_5_ with SCl_4_ under abstraction of a chloride ion [equation (6)].

SCl_4_ + UCl_5_ → (SCl_3_)[UCl_6_] [equation (6); Sawodny *et al.*, 1983 ▸].

## Refinement

Crystal data, data collection and structure refinement details are summarized in Table 3 ▸. Atomic coordinates of the previously reported (SCl_3_)[UCl_6_] structure (Sawodny *et al.*, 1983 ▸) were used for refinement. The structure was refined as an inversion twin with a twin ratio of 4.4:1. As a result of the similarity of the *a* and *b* lattice parameters, a fourfold twin was also considered; refinement of this twin model led to insignificant twin fractions. *R*_int_ for the tetra­gonal crystal system was above 0.4, ruling out a higher symmetry model.

## Supplementary Material

Crystal structure: contains datablock(s) I. DOI: 10.1107/S2414314620009608/wm4134sup1.cif

Structure factors: contains datablock(s) I. DOI: 10.1107/S2414314620009608/wm4134Isup2.hkl

CCDC reference: 2010898

Additional supporting information:  crystallographic information; 3D view; checkCIF report

## Figures and Tables

**Table 1:** Selected inter­atomic distances *d* (Å) for (SCl_3_)[UCl_6_] from the current and the previous refinement

Bond	*d* (current study)	*d* (Sawodny *et al.*, 1983 ▸)
U1—Cl1^ii^	2.5263 (15)	2.510 (10)
U1—Cl2^iii^	2.5297 (15)	2.531 (9)
U1—Cl3	2.5151 (16)	2.521 (10)
U1—Cl4	2.4869 (16)	2.485 (11)
U1—Cl5^i^	2.5045 (16)	2.499 (10)
U1—Cl6^ii^	2.5209 (15)	2.511 (9)
S1—Cl7	1.975 (2)	1.955 (14)
S1—Cl8	1.975 (2)	1.973 (13)
S1—Cl9	1.979 (2)	1.959 (13)

Symmetry codes: (i) −*x* + 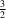 , −*y* + 1, *z* + 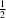 ; (ii) −*x* + 2, *y* − 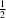 , −*z* + 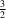 ; (iii) *x* + 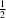 , −*y* + 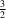 , −*z* + 2.

**Table 2:** Comparison of selected angles φ (°) for (SCl_3_)[UCl_6_] from the current and the previous refinement

Angle	φ (current study)	φ (Sawodny *et al.*, 1983 ▸)
Cl1^ii^—U1—Cl2^iii^	89.86 (5)	89.9 (3)
Cl3—U1—Cl6^ii^	179.02 (6)	179.3 (4)
Cl3—U1—Cl2^iii^	91.20 (5)	91.8 (4)
Cl3—U1—Cl1^ii^	89.76 (6)	90.0 (4)
Cl4—U1—Cl2^iii^	178.87 (6)	179.4 (4)
Cl4—U1—Cl1^ii^	89.44 (5)	89.6 (4)
Cl4—U1—Cl3	89.69 (6)	88.2 (4)
Cl4—U1—Cl5^i^	91.56 (6)	91.3 (4)
Cl4—U1—C6^ii^	90.83 (6)	91.2 (4)
Cl5^i^—U1—Cl1^ii^	178.95 (6)	179.0 (4)
Cl5^i^—U1—Cl6^ii^	90.89 (5)	90.1 (4)
Cl5^i^—U1—Cl2^iii^	89.14 (5)	89.2 (4)
Cl5^i^—U1—Cl3	89.92 (6)	89.5 (4)
Cl6^ii^—U1—Cl1^ii^	89.42 (5)	90.4 (4)
Cl6^ii^—U1—Cl2^iii^	88.28 (5)	88.7 (3)
Cl7^iv^—S1—Cl8	102.92 (10)	101.7 (7)
Cl7^i^—S1—Cl9	102.93 (10)	103.5 (7)
Cl8—S1—Cl9	102.21 (9)	101.8 (6)

Symmetry codes: (i) −*x* + 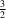 , −*y* + 1, *z* + 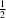 ; (ii) −*x* + 2, *y* − 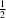 , −*z* + 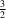 ; (iii) *x* + 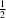 , −*y* + 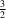 , −*z* + 2; (iv) *x* − 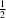 , −*y* + 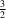 , −*z* + 2.

**Table 3:** Experimental details

Crystal data	
Chemical formula	(SCl_3_)[UCl_6_]
*M*_r_	589.14
Crystal system, space group	Orthorhombic, *P*2_1_2_1_2_1_
Temperature (K)	100
*a*, *b*, *c* (Å)	10.534 (2), 10.545 (2), 11.217 (2)
*V* (Å^3^)	1246.0 (4)
*Z*	4
Radiation type	Mo *K*α
μ (mm^−1^)	15.07
Crystal size (mm)	0.15 × 0.1 × 0.08

Data collection	
Diffractometer	Stoe IPDS 2T
Absorption correction	Numerical (*X-RED32*; Stoe 2016 ▸)
*T*_min_, *T*_max_	0.036, 0.090
No. of measured, independent and observed [*I* > 2σ(*I*)] reflections	18340, 3364, 3304
*R*_int_	0.046
(sin θ/λ)_max_ (Å^−1^)	0.686

Refinement	
*R*[*F* ^2^ > 2σ(*F* ^2^)], *wR*(*F* ^2^), *S*	0.019, 0.043, 1.08
No. of reflections	3364
No. of parameters	101
Δρ_max_, Δρ_min_ (e Å^−3^)	0.53, −1.01
Absolute structure	Refined as an inversion twin
Absolute structure parameter	0.186 (6)

Computer programs: *X-AREA* (Stoe, 2016 ▸), *SHELXL* (Sheldrick, 2015 ▸), *DIAMOND* (Brandenburg, 2019 ▸) and *publCIF* (Westrip, 2010 ▸).

## full crystallographic data

### Crystal data

**Table:**

(SCl_3_)[UCl_6_]	*D*_x_ = 3.141 Mg m^−^^3^
*M**_r_* = 589.14	Mo *K*α radiation, λ = 0.71073 Å
Orthorhombic, *P*2_1_2_1_2_1_	Cell parameters from 23552 reflections
*a* = 10.534 (2) Å	θ = 3.6–58.9°
*b* = 10.545 (2) Å	µ = 15.07 mm^−^^1^
*c* = 11.217 (2) Å	*T* = 100 K
*V* = 1246.0 (4) Å^3^	Block, dark yellow
*Z* = 4	0.15 × 0.1 × 0.08 mm
*F*(000) = 1044	

### Data collection

**Table:**

STOE IPDS 2T diffractometer	3364 independent reflections
Radiation source: sealed X-ray tube, 12 x 0.4 mm long-fine focus	3304 reflections with *I* > 2σ(*I*)
Planar graphite monochromator	*R*_int_ = 0.046
Detector resolution: 6.67 pixels mm^-1^	θ_max_ = 29.2°, θ_min_ = 2.7°
rotation method, ω scans	*h* = −14→13
Absorption correction: numerical (X-Red32; Stoe 2016)	*k* = −14→14
*T*_min_ = 0.036, *T*_max_ = 0.090	*l* = −15→15
18340 measured reflections	

### Refinement

**Table:**

Refinement on *F*^2^	Primary atom site location: other
Least-squares matrix: full	*w* = 1/[σ^2^(*F*_o_^2^) + (0.0148*P*)^2^ + 3.5607*P*] where *P* = (*F*_o_^2^ + 2*F*_c_^2^)/3
*R*[*F*^2^ > 2σ(*F*^2^)] = 0.019	(Δ/σ)_max_ = 0.001
*wR*(*F*^2^) = 0.043	Δρ_max_ = 0.53 e Å^−^^3^
*S* = 1.08	Δρ_min_ = −1.01 e Å^−^^3^
3364 reflections	Absolute structure: Refined as an inversion twin
101 parameters	Absolute structure parameter: 0.186 (6)
0 restraints	

### Special details

**Table:**

Geometry. All esds (except the esd in the dihedral angle between two l.s. planes) are estimated using the full covariance matrix. The cell esds are taken into account individually in the estimation of esds in distances, angles and torsion angles; correlations between esds in cell parameters are only used when they are defined by crystal symmetry. An approximate (isotropic) treatment of cell esds is used for estimating esds involving l.s. planes.
Refinement. Refined as a 2-component inversion twin.

### Fractional atomic coordinates and isotropic or equivalent isotropic displacement parameters (Å^2^)

**Table:**

	*x*	*y*	*z*	*U*_iso_*/*U*_eq_	
U1	0.97364 (2)	0.42494 (2)	0.98324 (2)	0.01499 (6)	
S1	0.42766 (14)	0.54654 (14)	0.91370 (13)	0.0181 (3)	
Cl1	0.82355 (14)	0.89033 (16)	0.63247 (14)	0.0239 (3)	
Cl2	0.59526 (14)	0.94785 (14)	0.86341 (13)	0.0225 (3)	
Cl3	0.93040 (17)	0.62131 (16)	0.86263 (15)	0.0273 (3)	
Cl4	0.85717 (15)	0.29644 (17)	0.83292 (15)	0.0258 (3)	
Cl5	0.72554 (14)	0.53738 (16)	0.59988 (14)	0.0241 (3)	
Cl6	0.97901 (16)	0.72883 (13)	0.39615 (13)	0.0220 (3)	
Cl7	0.92843 (16)	0.96521 (16)	0.91059 (14)	0.0250 (3)	
Cl8	0.60091 (14)	0.60665 (15)	0.87712 (15)	0.0241 (3)	
Cl9	0.32415 (15)	0.69986 (15)	0.88359 (15)	0.0255 (3)	

### Atomic displacement parameters (Å^2^)

**Table:**

	*U*^11^	*U*^22^	*U*^33^	*U*^12^	*U*^13^	*U*^23^
U1	0.01381 (8)	0.01650 (8)	0.01466 (9)	−0.00076 (7)	−0.00008 (7)	−0.00013 (7)
S1	0.0170 (6)	0.0191 (7)	0.0180 (6)	0.0009 (5)	−0.0012 (5)	0.0006 (5)
Cl1	0.0180 (6)	0.0306 (8)	0.0232 (7)	0.0019 (5)	0.0051 (5)	0.0038 (6)
Cl2	0.0221 (7)	0.0233 (8)	0.0221 (7)	0.0034 (5)	0.0020 (5)	−0.0045 (5)
Cl3	0.0355 (8)	0.0223 (7)	0.0241 (7)	0.0010 (6)	−0.0012 (7)	0.0060 (6)
Cl4	0.0219 (7)	0.0316 (8)	0.0239 (7)	−0.0038 (6)	−0.0057 (6)	−0.0059 (6)
Cl5	0.0170 (6)	0.0342 (8)	0.0211 (7)	0.0039 (6)	−0.0024 (5)	−0.0018 (6)
Cl6	0.0227 (6)	0.0192 (6)	0.0240 (6)	0.0011 (6)	−0.0028 (6)	−0.0027 (5)
Cl7	0.0273 (7)	0.0295 (8)	0.0182 (7)	−0.0015 (6)	−0.0004 (6)	0.0028 (6)
Cl8	0.0179 (6)	0.0283 (8)	0.0262 (7)	−0.0016 (5)	0.0012 (5)	0.0053 (6)
Cl9	0.0240 (7)	0.0243 (7)	0.0280 (8)	0.0073 (6)	−0.0025 (6)	0.0035 (6)

### Geometric parameters (Å, º)

**Table:**

U1—Cl4	2.4869 (16)	U1—Cl2^iii^	2.5297 (15)
U1—Cl5^i^	2.5045 (16)	S1—Cl7^iv^	1.975 (2)
U1—Cl3	2.5151 (16)	S1—Cl8	1.975 (2)
U1—Cl6^ii^	2.5209 (14)	S1—Cl9	1.979 (2)
U1—Cl1^ii^	2.5263 (15)		
			
Cl4—U1—Cl5^i^	91.56 (6)	Cl6^ii^—U1—Cl1^ii^	89.42 (5)
Cl4—U1—Cl3	89.69 (6)	Cl4—U1—Cl2^iii^	178.87 (6)
Cl5^i^—U1—Cl3	89.92 (6)	Cl5^i^—U1—Cl2^iii^	89.14 (5)
Cl4—U1—Cl6^ii^	90.83 (6)	Cl3—U1—Cl2^iii^	91.20 (5)
Cl5^i^—U1—Cl6^ii^	90.89 (5)	Cl6^ii^—U1—Cl2^iii^	88.28 (5)
Cl3—U1—Cl6^ii^	179.02 (6)	Cl1^ii^—U1—Cl2^iii^	89.86 (5)
Cl4—U1—Cl1^ii^	89.44 (5)	Cl7^iv^—S1—Cl8	102.92 (10)
Cl5^i^—U1—Cl1^ii^	178.95 (6)	Cl7^iv^—S1—Cl9	102.93 (10)
Cl3—U1—Cl1^ii^	89.76 (6)	Cl8—S1—Cl9	102.21 (9)

Symmetry codes: (i) −*x*+3/2, −*y*+1, *z*+1/2; (ii) −*x*+2, *y*−1/2, −*z*+3/2; (iii) *x*+1/2, −*y*+3/2, −*z*+2; (iv) *x*−1/2, −*y*+3/2, −*z*+2.
